# Spirituality and heart failure: a systematic review

**DOI:** 10.1007/s40520-023-02557-x

**Published:** 2023-09-22

**Authors:** Laura Cilona, Nicola Veronese, Diego Lalicata, Francesca Tantillo, Liliana Naro, Ligia J. Dominguez, Mario Barbagallo

**Affiliations:** 1 Department of Health Promotion, Maternal and Infant Care, Internal Medicine and Medical Specialties, Geriatric Unit, “G. D’Alessandro”– PROMISE – University of Palermo, Via del Vespro, 141, 90127 Palermo, Italy; 2 School of Medicine and Surgery, University Kore of Enna, Enna, Italy

**Keywords:** Heart failure, Spirituality, Religion, Quality of life, Depression

## Abstract

**Objectives:**

Heart failure (HF) is a frequent chronic disease with disturbing symptoms and complex treatments, associated with depression and lower quality of life. Some studies have shown that spirituality and religiosity may be relevant in these patients. We aimed to systematically review the medical literature on spirituality and religiosity in patients with HF.

**Methods:**

Major databases for studies investigating the effect of spirituality and religiosity in people affected by HF were searched from inception until 26th April 2023. Studies with clear definition of spirituality or religiosity, validated diagnosis of HF, and reporting outcomes of interest (i.e., incidence of mortality, cardiovascular outcomes, and quality of life) were included.

**Results:**

Among 810 non-duplicate records, we screened the full texts of 25 works. After excluding 18 studies, we included 7 studies (3 observational and 4 interventional) comprising 1234 HF patients followed up over a median of 3 months. Definitions of spirituality and religiosity were heterogeneous among the studies. The intervention studies showed improvements in quality-of-life parameters, some cardiovascular outcomes, or mortality, and the observational studies showed significant associations with these outcomes.

**Conclusions:**

Despite the extreme heterogeneity of the populations included, of the definition of spirituality and religiosity, and of the interventions in the few studies that included it, all the studies reported some positive associations with the outcomes examined. Spirituality/religiosity is an aspect not generally taken into account in the usual practice of medicine and can potentially contribute to improving the conditions of patients with HF, a chronic disease with unfavorable prognosis.

**Supplementary Information:**

The online version contains supplementary material available at 10.1007/s40520-023-02557-x.

## Introduction

Heart failure (HF) was called a new epidemic at the end of the 1990s [1] proposed to be related not only to an increase in its incidence but also to a greater survival and hospitalization rates of patients diagnosed with heart failure [2]. Today, HF is among the most prevalent chronic diseases, with estimates in the US of over 6.5 million people living with this pathological condition [3]. Worldwide, 2019 estimates resulted in 5.05 million years of life lost due to disability (YLDs) across the world due to HF, with an age-standardized rate of 711.90 per 100,000 population [4]. Nearly 42% of persons diagnosed with HF die within 5 years [3], and its incidence and prevalence is expected to increase due to aging of the population, reduced mortality due to myocardial infarction, and more effective treatments of HF prolonging survival with damaged heart function [5].

People with HF need complex treatment regimens to manage the constantly present and disturbing symptoms, such as dyspnea and peripheral edema, with instability in fluid management and sodium intake, need of frequent monitoring and hospitalizations [3], which worsen quality of life and is frequently associated with depressive symptoms [6]. The high rates of depression in patients with HF are not only an adverse outcome itself but also they are associated with higher mortality risk [7].

For some years, studies have shown how factors related to spirituality and religiosity in their diverse expressions may be relevant in patients with HF [8], especially when the disease worsens over time [9]. Several studies have shown that these aspects can be related to the presence of depression, and to the perception of better psychological well-being and quality of life in these patients [10, 11]. Most of the studies available in the literature include small groups and are heterogeneous.

Given this background, with this systematic review, we aimed to determine the importance of spirituality and religiosity in patients with HF found in the medical literature in terms of promotion of psychological well-being, depression, and quality of life.

## Materials and methods

This systematic review adhered to the PRISMA statement [12] and followed a pre-planned, but unpublished protocol that can be requested by contacting the corresponding author.

### Data sources and searches

Four investigators (LC, LN, FT, DL) independently conducted a literature search using PubMed/Medline, Embase, Web of Science from database inception until 26th April 2023, for studies investigating the effect of religiosity in people affected by HF.

In PubMed/Medline, the following search strategy was used: “(spirituality OR religion OR Spiritual Coping OR Religiosity Coping) AND (heart failure OR Cardiac Failure OR Myocardial Failure)”, adapting the search for the other databases. Any inconsistencies during title, abstract, and finally full-text screening were resolved by consensus, with a third senior author (LD).

### Study selection

Inclusion criteria for this systematic review were: (i) clear definition of spirituality or religiosity; (ii) validated diagnosis of HF (e.g., medical records, echocardiographic data); and (iii) reporting outcomes of interest. Studies were excluded if: (i) mixed patients with HF with other conditions; and (ii) not human beings. Conference abstracts were included only if they contained all the data required for the systematic review.

### Data extraction

One investigator (LC) extracted key data from the included articles in a standardized Excel spread sheet, with a second independent investigator (NV) checking the data. For each article, we extracted the data on authors’ names, year of publication, country, condition, sample size, age and percentage of females, diagnostic criteria used for the definition of HF (including its severity), and time of follow-up.

### Outcomes

The primary outcomes were the incidence and risk of quality of life, mortality, and cardiovascular outcomes in patients affected by HF.

### Data synthesis and analysis

The data are reported descriptively since extremely heterogeneous definitions of spirituality or religiosity were used in the studies. We, therefore, reported the main findings of the studies included indicating if the associations reported were statistically significant at a p-value < 0.05.

## Results

### Literature search

**Fig. 1:**
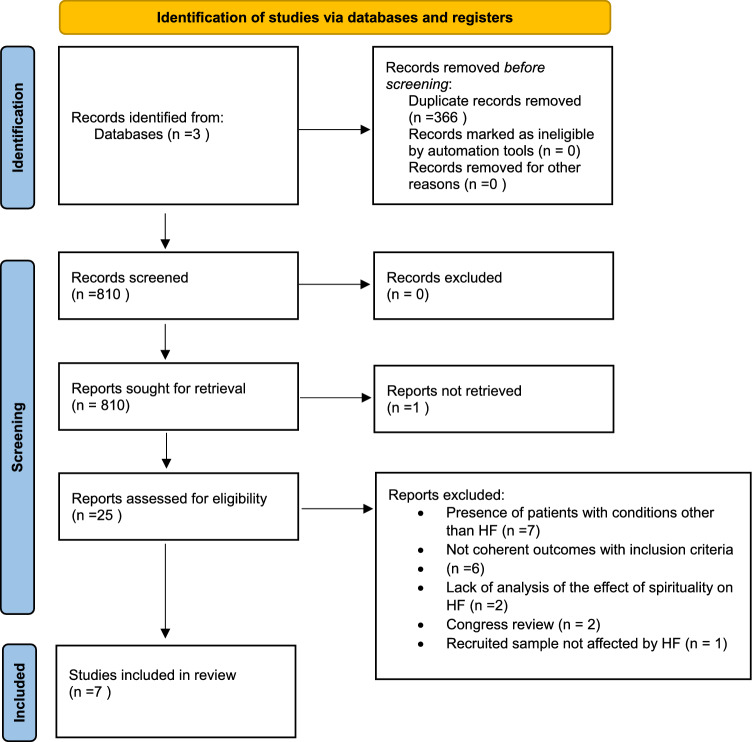
PRISMA flowchart

Figure 1 shows the literature search made for this systematic review. Among 810 non-duplicate records, we screened the full texts of 25 works. Supplementary Table 1 reports the details of excluded studies. After excluding seven studies, since they included patients with conditions other than HF, other six since they did not include outcomes of interest, two since they did not report the effect of spirituality on HF, two conference abstracts, and one that recruited sample not affected by HF, we included seven studies [13–19] in this systematic review.

### Descriptive characteristics

As shown in Table 1, the seven studies included a total of 1,234 patients with HF, with a mean age of 65.8 years, and followed up over a median of 3 months. Six studies were made in the US, while one in Iran. There were no studies from Europe that met the inclusion criteria of this systematic review. Three of the included studies were observational [14–16] and four studies involved interventions [13, 17–19]. The definition of spirituality was extremely heterogeneous including spirituality health, a multicomponent intervention, religious attitudes, inner peace and harmony, spiritual peace and counseling, and an optimized protocol for Jehovah’s Witnesses undergoing complex heart surgery. The diagnosis of HF was made using medical records in the majority of the studies, mainly in moderate to severe HF (NYHA classes III–IV) (Table 1).

**Table 1:** Descriptive characteristics of the studies included

Authors, year	Mean age	Country	Length of follow-up (months)	Study design	Definition of spirituality	Total sample size	Diagnostic criteria for HF	Severity of HF and other characteristics of HF
Abdi, 2018 [13]	73.7	Iran	1.5	Clinical controlled trial	Spirituality health (SH) (health in beliefs, opinions, moral values and practice) tested with religion-spiritual program	93	Medical records	Not specified
Hooker, 2016 [19]	61.6	USA	3	Clinical controlled trial	multicomponent intervention (Education, Reflection, Activity Spirituality)	66	Medical records	Not specified
Park, 2014 [16]	66.7	USA	3	Longitudinal study	Forgiveness, Daily spiritual experience, Belief in afterlife, Religious identity, Religious social support, Public religious practices, Positive religious coping, Inner peace and harmony, spiritual counseling	212	Medical records	Severe CHF (NYHA classes III–IV)
Park, 2016 [14]	68.8	USA	60	Longitudinal study	Inner peace and harmony	321	Medical records (echocardiogram, MUGA, other nuclear tests of ventricular function)	Left-sided, systolic congestive heart failure of at least moderate severity (NYHA classes I–II) with the ejection fraction (EF) < 45, ineligible for transplantation
Park, 2020 [15]	68.6	USA	6	Longitudinal study	Spiritual peace	382	Medical records (echocardiogram, MUGA, other nuclear tests of ventricular function)	Left-sided, systolic congestive heart failure of at least moderate severity, ejection fraction (EF) < 45, ineligible for transplantation
Tadwalkar, 2014 [17]	57.4	USA	3	Pilot study, Randomized controlled trial	Spiritual counseling	23	Assessment of the admitting physician	(NYHA) classes III–IV, diagnosis of congestive heart failure for more than 3 months
Tanaka, 2015 [18]	64	USA	192	Retrospective	Jehovah’s witness	137	Patient charts and the institutional cardiac surgery outcomes database	NYHA class IV and emergency/urgent cases
TOTAL		Six studies in USA, one in Iran	Median = 3 months	Four studies observational, three intervention		1234	Five studies medical records, other tools in two studies	

### Main findings

Table 2 indicates the main findings of our systematic review. In one study including 66 patients with HF, over a follow-up period of 3 months, a multicomponent intervention (made of Education, Reflection, Activity, and Spirituality) improved the quality of life compared to standard care [19]. A retrospective study among 137 Jehovah’s Witness with severe HF reported that a preoperative optimization protocol including discontinuing antiplatelets and adding iron/vitamin or erythropoietin to achieve hemoglobin concentration > 12 g/dL before complex cardiovascular surgery resulted in decreased mortality and HF vs. unoptimized protocol [18]. Moreover, in one study including 212 patients affected by severe HF, daily spiritual experience was linearly associated with a significantly less functional limitation due to HF [16]. Furthermore, inner peace and harmony were associated with a lower risk of mortality compared to patients not following these indications [14]. Finally, two studies (one observational and one small pilot study) reported that spirituality was associated with a significantly better quality of life [15, 17].

**Table 2:** Effect of spirituality on quality of life, cardiovascular outcomes, and mortality in patients with heart failure

Type of spirituality	Quality of life	CVD outcomes	Mortality
Multicomponent intervention (education, reflection, and activity spirituality)	Improved	Not available	Not available
Jehovah’s witness	Not available	Significantly higher prolonged ventilation, acute renal failure	Lower in Jehovah’s witness with an optimized protocol vs. controls in unoptimized protocol
Daily spiritual experience	Not available	Significant linear correlation with less functional limitation due to heart failure	Not available
Inner peace and harmony	Not available	Not available	Lower risk in patients with higher inner peace and harmony
Spiritual counseling	Improved	Not available	Not available
Spirituality health (SH) (health in beliefs, opinions, moral values and practice) tested with religion–spiritual program	Improved	Not available	Not available

## Discussion

This systematic review on the association of spirituality or religiosity with quality of life, mortality, and cardiovascular outcomes in patients with HF, despite the extreme heterogeneity of the populations included, of the definition of spirituality and religiosity, and of the interventions in the few studies that included it, showed that in all the included studies there were some positive associations with the outcomes examined. This dimension—spirituality/religiosity—is an aspect that is not generally taken into account in the usual practice of medicine and that can potentially contribute to improving the conditions of patients with HF, a chronic condition with a still very unfavorable prognosis.

In recent decades, interest in considering spiritual care and religiosity as part of the determinants of health has grown. This is particularly true when considering incurable, chronic, or terminal illnesses [20, 21]. Research and professional healthcare clinical guidelines now more often include the spiritual needs of patients as a fundamental holistic component in ideal health care assessment. Excluding the positive value of these human dimensions can lead to a poor and incomplete vision of the determinants of human health [22, 23]. Avoiding exposure to conventional risk factors for the development and progression of diseases such as inadequate nutrition, physical inactivity, environmental pollution, smoking, poor quality sleep, as well as receiving an adequate clinical care is fundamental. However, other behavioral and social factors cannot be neglected being part of what essentially shapes human health. These factors, for example, may help to explain why some persons are more resilient and better cope with living with a chronic illness in adverse circumstances, while others are not [24, 25].

There is increasing evidence showing how diverse social, psychological, and environmental factors strongly influence physical and mental health, sometimes with magnitudes comparable to those observed for conventional risk factors. For example, participation in religious communities has been associated with better health outcomes such as reduced mortality, depression, substance abuse, and suicide [26–28].

In reference to HF, this is a complex syndrome and the progression and death due to this condition can be a slow and very difficult process. It frequently means severe deterioration and an uncertain period of suffering for patients as well as for their caregivers, which can generate important challenges from the spiritual and psychosocial point of view [8, 15, 29]. Saunders coined the term “total pain” referring to the experience of chronic HF that includes spiritual pain in which there is loss of personal integrity and lack of inner peace [30]. The spiritual needs of HF patients and their caregivers have indeed been characterized by isolation, hopelessness, and altered self-image associated with chronic illness and disability [8].

Among the studies that met the requirements to be included in our systematic review, four were intervention studies [13, 17–19]. Despite the interventions were thoroughly dissimilar, which did not allow further statistical analyses of the results, all four studies resulted in improvements of quality-of-life indices and/or some cardiovascular and mortality outcomes. In a pilot study, Taldwalkar et al. [17] tested the effect on quality-of-life outcomes in 23 patients with HF of an adjunct spiritual counseling (“religious” or “non-religious” based strictly on their personal preferences) performed daily or once every 2 days during hospitalization. Quality-of-life scores completed at baseline, at 2 weeks, and at 3 months improved in time with the adjunct spiritual counseling suggesting that the addition of this intervention to standard medical management for patients with HF seems to have a positive impact on quality of life. Tanaka et al. [18] retrospectively examined the effects of a preoperative optimization protocol for 144 Jehovah’s Witnesses undergoing complex cardiovascular surgery, including discontinuing antiplatelets and adding iron/vitamin or erythropoietin to achieve a target hemoglobin greater than 12 g/dL vs. unoptimized groups. Hospital mortality was 2.2% vs. 15.9% in optimized vs. unoptimized protocol, respectively, and a composite outcome (including HF) was observed in 22.6% and 50.0% of optimized vs. unoptimized protocol, respectively. In clinical practice, the difficulty in managing a Jehovah’s Witness patient who needs a blood transfusion and who utterly rejects it is well recognized. This study showed that preventive actions guaranteeing an adequate hemoglobin level can significantly change the mortality risk of these patients undergoing complex medical interventions. Hooker et al. [19] evaluated the efficacy of a resource-sparing psychospiritual intervention (12-week mail-based intervention addressing spirituality, stress, and coping) to improve quality of life in 33 HF patients. Participants who completed the intervention reported evidence suggesting increased quality of life, decreased depressive symptoms, and less searching for meaning, rating the intervention as acceptable and beneficial. Most participants believed spirituality should continue to be included, although they disagreed on the extent to which religion should remain. These results suggest that clinicians might be open to issues of spirituality as they may relate to quality of life in patients with HF. Finally, the study by Abdi et al. [13] was conducted to determine the effect of religion intervention on life satisfaction and depression in Iranian older adults with HF. Patients were randomly allocated into experimental (*n* = 46) and control (*n* = 47) groups. The intervention was a religion–spiritual program based on the Richards and Bergin model, according to Islam and Shia regulations, and conducted during six sessions, each 30–45 min. For participants in the intervention group, life satisfaction index-Z and Beck depression inventory scores were improved compared to the control group.

As regards the three observational studies, all were conducted by the group of Park et al. [14–16]. One of the studies [16] evaluated seven dimensions of religion/spirituality (R/S) and three dimensions of well-being in a sample of 111 patients with advanced HF at baseline and 3 months later. Forgiveness was related to less subsequent depression, while belief in afterlife was related to poorer mental health. The results showed that R/S may not affect physical well-being but may have potent influences on other aspects of well-being, particularly existential aspects. The second study by Park et al. [14] explored the association of spirituality with mortality risk in 191 HF patients, of whom 32% died during the study period. Although both religion and spirituality were associated with better health behaviors at baseline, a proportional hazard model showed that only spirituality was significantly associated with 20% lower mortality risk, controlling for demographics, health status, and health behaviors. Experiencing spiritual peace, along with adherence to a healthy lifestyle, was a better predictor of mortality risk than physical health indicators such as functional status and comorbidity. Finally, a recent study by the same group [15] examined whether R/S may mitigate depressive symptoms and declining quality of life with disease progression beyond provision of social support in HF patients at baseline and 6 months later. Controlling for demographics and baseline health status, higher levels of spiritual peace and social support each uniquely predicted increased positive states of mind; only social support predicted improved physical health-related quality of life; neither spiritual peace nor social support predicted change in mental health-related quality of life; and only spiritual peace predicted reduced levels of depressive symptoms across 6 months. These results suggest that R/S may play an important role distinct from social support in promoting well-being in people with HF.

### Strengths and limitations

The strength of our systematic review is that, to the best of our knowledge, this is the first study to explore whether spirituality/religiosity can have important effects on quality of life and health outcomes in patients with HF. The limitations of the review are mainly due to the few studies available to date addressing these issues (for example, no European studies met the inclusion criteria), and, above all, to the extraordinary heterogeneity of the designs, definitions, and interventions found in the seven studies that we were able to include in the systematic review, which did not allow us to perform further statistical analyses. However, and despite this profound heterogeneity, all the studies showed the importance of these dimensions that are generally not taken into account in the clinical practice of these complex patients with great health and spiritual needs.

## Conclusions

Among four intervention and three observational studies included in this systematic review, spirituality and religiosity seem to play a fundamental role in contributing to improving quality of life, reducing depression, and improving some cardiovascular and health outcomes in patients with HF. Our results, in spite of the limitations, emphasize and draw attention to the importance of human dimensions that are frequently overlooked in usual clinical practice in patients with a complex, chronic, progressive, and often fatal pathological condition. Clinicians should be aware and consider spirituality/religiosity to help improving quality of life in the holistic care of these patients.

### Supplementary Information

Below is the link to the electronic supplementary material.

Supplementary file1 (DOCX 13 KB)
