# Supplementary material for: Spirituality and heart failure: a systematic review
Source: Aging Clin Exp Res. 2023 Sep 22;35(11):2355–61. doi: 10.1007/s40520-023-02557-x (PMC10627967; doi:10.1007/s40520-023-02557-x)
Supplement: Supplementary file 1 — Supplementary file1 (DOCX 13 KB) [file 40520_2023_2557_MOESM1_ESM.docx]

**Supplementary Table 1. References excluded, at full-text revision, with motivations.**

**A)    Presence of patients with conditions other than HF (n =7):**

  - Religion, risk, and medical decision making at the end of life

  - Religion and Remission of Depression in Medical Inpatients

  - Spiritual well-being in patients with advanced heart and lung disease

  - Addressing Patient Emotional and Existential Needs During Serious Illness Results of the Outlook Randomized     Controlled Trial

  - Current measures of distress may not account for what's most important in existential care interventions Results     of the outlook trial

   - Exploring the spiritual needs of people dying of lung cancer or heart failure a prospective qualitative interview study of patients and their carers

- The Impact of Spiritual Care Education on Anxiety in Family Caregivers of Patients with Heart Failure

**B)    Not coherent outcomes with inclusion criteria  (n =6)**

- Acute heart failure presentations and outcomes during the fasting month of Ramadan an observational report from seven Middle Eastern countries

- Effect of Spiritual Care Program on Resilience in Patients with Heart Failure: A Randomized Controlled Clinical Trial

- Fasting during the month of Ramadan among patients with chronic kidney disease Renal and cardiovascular outcomes

- Religious beliefs and well-being and distress in congestive heart failure patients

- Religious struggle as a predictor of subsequent mental and physical well-being in advanced heart failure patients

- How Does Spiritual Well-Being Change Over Time Among US Patients with Heart Failure and What Predicts Change?

**C)    Lack of analysis of the effect of spirituality on HF (n =2)**

- Estimated longevity and changes in spirituality in the context of advanced congestive heart failure

- Realization and personalization by facing fatality. A grounded theory of developing the view of dying in people with heart failure

**D)    Congress review (n = 2)**

-       Religious beliefs towards the end of life among patients with chronic heart failure

-       Psychological symptoms and religiosity in patients with ventricular assist devices: A cohort study
